# Identification of a Novel Mutation in a Pseudohypoparathyroidism Family

**DOI:** 10.1155/2011/509549

**Published:** 2011-07-21

**Authors:** Zhi-Min Miao, Can Wang, Bin-Bin Wang, Dong-Mei Meng, Dong-Mei Su, Zhi Cheng, Qiao-Lian Wen, Lin Han, Qing Yu, Xu Ma, Chang-Gui Li

**Affiliations:** ^1^Gout Laboratory, The Affiliated Hospital of Qingdao University Medical College, 16 Jiangsu Road, Qingdao 266003, China; ^2^Graduate School, Peking Union Medical College, Beijing 100081, China; ^3^Center of Genetics, National Research Institute for Family Planning, Beijing 100081, China; ^4^Center of Genetics, World Health Organization Collaborating Centre for Research in Human Reproduction, Beijing 100081, China

## Abstract

Pseudohypoparathyroidism type Ia (PHP Ia) is defined as a series of disorders characterized by multihormone resistance in end-organs and Albright hereditary osteodystrophy (AHO) phenotype. PHP Ia is caused by heterozygous inactivating mutations in GNAS, which encodes the stimulatory G-protein alpha subunit (Gsa). A patient with typical clinical manifestations of pseudohypoparathyroidism (PHP) (round face, short stature, centripetal obesity, brachydactyly, and multi-hormone resistance: parathyroid hormone (PTH), thyroid-stimulating hormone (TSH), and gonadotropins) presented at our center. The sequence of the GNAS gene from the patient and her families revealed a novel missense mutation (Y318H) in the proband and her mother. An in vitro Gsa functional study showed that Gsa function was significantly impaired. These results stress the importance of GNAS gene investigation.

## 1. Introduction

PHP Ia and pseudo-pseudohypoparathyroidism (PPHP) are disorders caused by heterozygous inactivating mutations in GNAS, which encodes Gsa, and usually coexist in the same family [1, 2]. Paternal transmission of GNAS mutations only lead to the AHO phenotype, which is characterized by a round face, short stature, centripetal obesity, brachydactyly, Subcutaneous (sc) ossifications, mental deficits, or developmental delay, and is termed PPHP. Maternal transmission leads to AHO plus hypocalcemia, hyperphosphatemia, and multi-hormone resistance, such as to PTH, TSH, gonadotropins, and growth hormone releasing hormone (GHRH), and is termed PHP Ia [1, 3–6].

The GNAS gene is located on chromosome 20q13.11 and consists of 13 exons and 12 introns [1]. GNAS mutations have been found throughout the coding sequence of the gene [4, 7], and about 35% of all mutations described are within exon 7. Many authors have demonstrated the tissue-specific imprinting nature of Gsa [8]. In PHP Ia patients, GNAS is biallelically expressed in most tissues, in which Gsa activity was reduced by approximately 50% because of a reduction of Gsa mRNA and protein expression [9]. While in few other tissues such as renal proximal tubules, the thyroid, the gonads, and the pituitary, GNAS is maternally expressed because of the suppression (imprinting) of the paternal allele [4, 10]. This might explain why the multi-hormone resistance in PHPIA patients primarily involves four hormones: PTH, TSH, gonadotropins, and GHRH [4, 11], all of which stimulate Gs-coupled pathways in their target tissues.

Here we reported a PHP family and a novel missense mutation (c.952 T>C, Y318H) in the GNAS gene of this family. 

## 2. Patients Presentation

### 2.1. Cases

**Figure 1:**
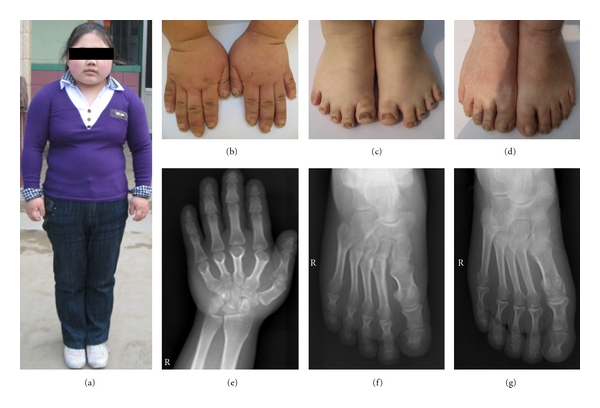
(a) The proband appeared short, obese, and face round. (b) The proband's hands. (c) The proband's feet. (d) Feet of the proband's mother. (e) An X-ray of patient's right hand showing that her metacarpals and phalanges are generally shortened. The both ends of the twice-fifth metacarpals are enlarged. The first distal phalanx, twice-fifth middle phalanges and twice-fifth distal phalanges are thickened. (f) The X-ray of the patient's right foot showing that her metatarsus and phalanges are generally shortened, especially the first metatarsus and phalanx. The distal ends of the first metatarsus, first phalanx, and twice-fifth phalanges are enlarged. The proximal ends of the first phalanx and twice-fifth phalanges are widened. (g) The feet of the patient's mother looked normal; however, an X-ray of her right foot showed that she had a similar, but less severe, phenotype to her daughter.

The female proband was the product of a normal-term delivery and was the second child of her family. Her birth weight and length were in the normal range, except for abnormal weight gain and short stature, no further atypical signs were observed in childhood. She underwent her first menstruation at the age of 15, but the menstrual cycle was about 6 months and was accompanied by oligomenorrhea. In addition, her breast remained undeveloped. She attended our hospital for breast development retardation and irregular menstruation at the age of 22. At this time, she showed features of AHO: round face, short stature, and overweight (65 kg, height 142 cm), resulting in an abnormal body mass index (32.25 kg/m^2^) (Figure 1(a)). In addition, she also showed brachymetacarpia (Figures 1(b) and 1(e)) and brachymetatarsia (Figures 1(c) and 1(f)). 

Her mother showed features of slight AHO, such as a round face, short stature, and slight brachymetatarsia (Figure 1(g)). Her weight was 46 kg, and her height was 148 cm, resulting in a normal body mass index (21 kg/m^2^). 

Biochemical details of the patient revealed apparent elevated PTH, serum calcium in the midnormal range, and normal serum phosphate. She also displayed multihormone resistance, characterized by elevated plasma TSH and decreased plasma Estradiol (E2). The growth hormone (GH) level and islet function test results were normal. The patient's mother did not show any sign of hormone resistance (Table 1). 

The parents gave informed consent to further investigations of all family members.

### 2.2. Sequencing Analysis

**Figure 2:**
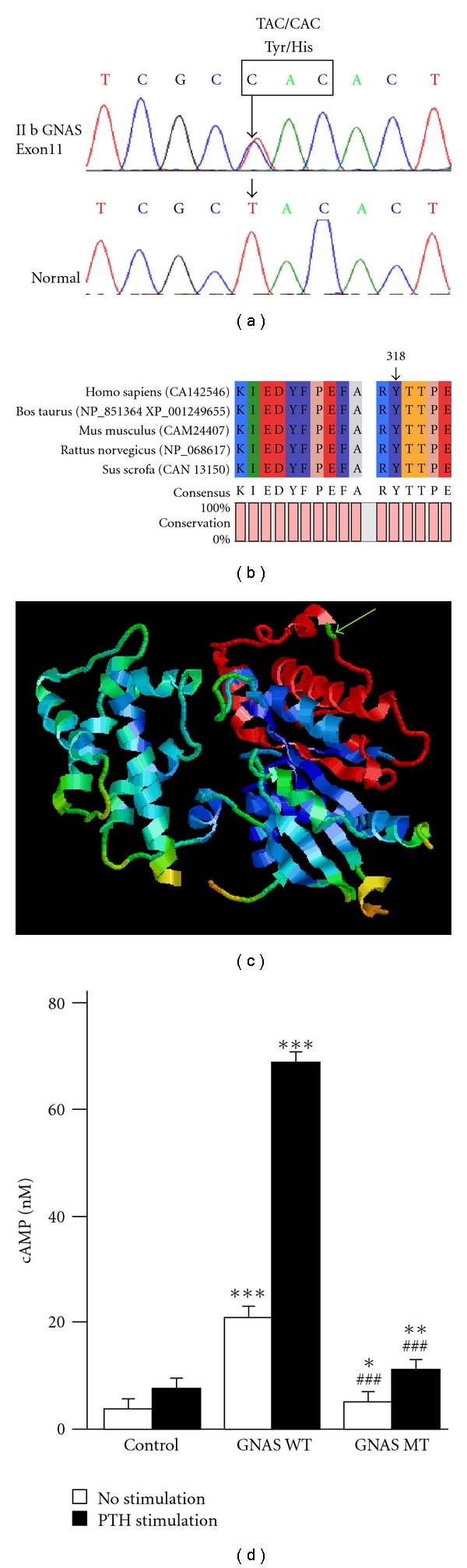
(a) Sequencing analysis of exon 11 in patient II b. The figure shows that the missense mutation at codon 318 results in a Tyr to His substitution. The same mutation was found in the proband's mother, who is affected by PPHP. (b) Gsa conservation analysis with CLC free, the result shows that Gsa codon 318 is in the highly conserved region. (c) The tertiary structure of Gsa, *α*4-*β*6 loop was shown in red, and Tyr-318 residue was the green point indicated by green arrow. (d) A cAMP assay was used to assess the Gsa function of GNAS WT and GNAS MT, with or without stimulation with PTH 1–34 (10^−8^ M) in OK cells. *P < 0.05; **P < 0.01; ***P < 0.001 (compared with control). ^###^ *P* < 0.001 (compared with GNAS WT).

**Figure 3:**
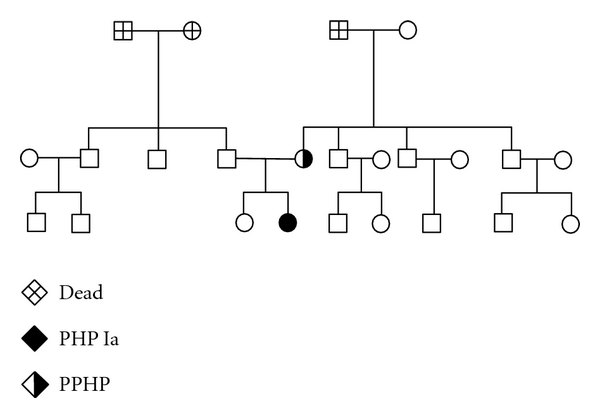
Family tree of the patient.

Direct sequencing of the amplified GNAS genomic DNA fragments revealed a heterozygous missense mutation within exon 11 (c.952 T>C) (Figure 2(a)) in the proband. The T>C transversion results in a Tyr to His substitution at codon 318 (Y318H, NP_000507.1). To the best of our knowledge, this is a novel mutation in GNAS (http://www.HGMD.cf.ac.uk/ac/index.php). This mutation was also found in patient's mother, who was affected with PPHP. No mutation was detected in other family members (Figure 3) and 50 healthy individuals. And the mutation has been confirmed on second PCR products.

### 2.3. In Vitro Gsa Biological Activity Analysis

Functional Gsa can stimulate adenylyl cyclase, which can catalyze the synthesis of cAMP from ATP within cells [4]. To determine whether this is a loss of function mutation, OK cells were transfected with pEGFP-N1 (as a control), pEGFP-N1-GNAS WT (wild type), and pEGFP-N1-GNAS MT (mutant type), respectively, and stimulated with PTH 1-34. OK cells were chosen because of their endogenous expression of PTH receptor type 1 [12]. The results showed that compared with GNAS WT, though the GNAS MT can still enhance cAMP accumulation in OK cells (GNAS WT compared with control: 26.32 ± 1.29 nM versus 3.69 ± 1.84 nM, *P* < 0.01 and GNAS MT compared with control: 5.26 ± 1.36 nM versus 3.69 ± 1.84 nM, *P* < 0.05.  Figure 2(d)), GNAS MT's function was significantly impaired, either when treated with PTH or untreated (GNAS MT compared with GNAS WT: untreated with PTH 5.26 ± 1.36 nM Versus 26.32 ± 1.29 nM, *P* < 0.001; treated with PTH 13.96 ± 1.79 nM versus 68.92 ± 1.79 nM, *P* < 0.001.  Figure 2(d)) [12]. The identification of a GNAS mutation and reduced Gsa activity confirmed the presumptive diagnosis of PHPIa and PPHP.

## 3. Discussion

By direct sequencing analysis of proband's GNAS gene we discovered a heterozygous missense mutation within exon 11 (c.952 T>C), though there were many mutations that have been reported before (http://www.HGMD.cf.ac.uk/ac/index.php). This mutation is novel, thus expanded the spectrum of GNAS mutation associated with PHP and PPHP. Besides it is the first missense mutation of GNAS detected in Chinese Han PHP family. This mutation in exon 11 results in a Tyr to His substitution at codon 318 (Y318H, NP_000507.1). Gsa codon 318 is in the highly conserved region (Figure 2(b)), which is considered to have more important biological significance. Measurement of cAMP in wild-type and Y318H mutant GNAS transfected OK cells demonstrated that the missense mutation could significantly impair Gsa function. This was roughly consistent with previously reported 50% reductions of Gsa function in PHPIa and PPHP [9].

GNAS has been termed one of the most complex gene loci in the human genome [7]. Moreover, several different transcript variants from GNAS have been described, and two long (Gsa-L) and two short (Gsa-S) transcript variants are created, by differential splicing of exon 3 and/or use of two 5′ splice sites of exon 4. These variants contain alternatively spliced exon 3 and/or a CAG sequence, respectively [7, 13]. Each of these variants is capable of stimulating both adenylate cyclase and calcium channels and can represent the transcript for functioning proteins [14]. In addition, these variants are regarded to be functionally nearly identical. Gsa-L and Gsa-S were also shown to be biochemically nearly indistinguishable in a mammalian cell line that lacks endogenous Gsa [15]. Therefore, we selectively constructed a typical long type of Gsa (ALEX GNASL, 394aa) as a representative. G protein is integral component of various signaling pathways; it consists of *α* and *βγ* subunits. Inactivated Gsa is integrated with *βγ* subunits; the comparison of structure of Gsa with that of inhibitory G-protein alpha subunit (Gia) suggested that the *βγ* binging surface of Gsa is strictly conserved in sequence and structure to that of Gia, except for the carboxyl-terminal helix and the *α*4-*β*6 loop, that may mediate receptor specificity [16], as is showed in Figure (Figure 2(c)). Our patient's mutation in residue Tyr-318 is located within the *α*4-*β*6 loop. Thus we speculate that the reason for this missense mutation in our patients resulting in a partial deficiency (50%) of Gsa is possibly that the exchange of Tyr to His in Gsa affected the binding of Gsa with *βγ*. However, the precise mechanism causing loss of function of Tyr 318 His mutant Gsa needs farther study.

GNAS has the feature of genomic imprinting [14], transmission of GNAS mutations through the maternal germline results in PHP Ia, whereas inheritance from the father causes only AHO [4]. We sequenced GNAS gene of proband and her families, but only examined mutation (Y 318 H) in proband and her mother. Considering variation of phenotype due to genomic imprinting [8], it is easily to understand patient's genotype and phenotype of PHP Ia. One reasonable explanation for patient's mother's genotype and slight AHO is that the mutation came from germ cell offered by her father, but it was impossible to validate. Overall, the pattern of transmission of this novel heterozygous mutation is consistent with the general model proposed for PHP.

## 4. Conclusion

Although many mutations that can impair Gsa function have been identified in GNAS, the c. 952 T>C missense mutation is novel, and it is the first missense mutation of GNAS detected in Chinese Han PHP family. The identification of this mutation contributes to the understanding of the genotype of PHP and stressed the importance of GNAS gene investigation in diagnosis of PHP.

##  Conflict of Interests Disclosure

The authors declare no conflict of interests.

## Figures and Tables

**Table 1:** Blood biochemical and hormonal analysis.

	I b	II b	Normal value
Serum Calcium	2.56	2.39	2.0–2.8 mmol/L
Serum Phosphate	1.1	1.51	0.8–1.6 mmol/L
Plasma PTH	27.52	*152.4*	15–65 pg/mL
Plasma GH	4.216	4.763	<5 ng/mL
Plasma TSH	3.73	*14.56*	0.27–4.2 uIU/mL
Plasma Free T3	3.53	3.18	3.1–6.8 pmol/L
Plasma Free T4	18.23	*8.47*	12–22 pmol/L
Plasma LH	Normal	6.67	2.4–12.6 mIu/mL
Plasma FSH	Normal	11.34	3.5–12.5 mIu/mL
Plasma Estradiol	Normal	*23.34*	24.5–195 pg/mL
Plasma INS	13.34	15.29	2.6–24.9 uIU/mL

Values in italics represent means outside the reference range; I b represented patient's mother; II b represented the patient.
